# Utilisation of postnatal care services among maternal in Maeen District – Sana’a City, Yemen

**DOI:** 10.1186/s12884-024-06617-6

**Published:** 2024-06-13

**Authors:** Abdulnasser A. Haza’a, Marzoq A. Odhah, Saddam A. Al-Ahdal, Fawz M. Abol–Gaith, Nada A. Ismail, Mohammed S. Al-Awar, Abdulfatah S. Al-Jaradi, Wedian S. Eidah, Manar M. Kaid

**Affiliations:** 1 Nursing and Midwifery Department, Faculty of Medicine and Health Sciences, Al-Razi University, Sana’a, Yemen; 2 Department of Nursing, Faculty of Medicine and Health Sciences, Sana’a University, Sana’a, Yemen; 3 Nursing and Midwifery Department, Faculty of Medical Sciences, Azal University, Sana’a, Yemen; 4 Department of Laboratory, Al-Razi University, Sana’a, Yemen

**Keywords:** Factors, Utilisation, Postnatal care, Maternal health

## Abstract

**Background:**

One of the most effective ways to reduce maternal and neonatal mortality is to improve mother and newborn health via the provision of appropriate postnatal care services by qualified healthcare providers. However, there is limited information on the use of postnatal care services in Yemen. This study aimed to determine the utilisation of postnatal care services among mothers in Yemen.

**Methodology:**

A descriptive cross-sectional study was conducted in the Maeen District of Sana’a City, Yemen from December 2022 to January 2023. Convenience sampling was employed to recruit 321 participants. Semi-structured questionnaires were applied as the study tool in the face-to-face survey.

**Result:**

Less than half (45.2%) of the study participants utilised postnatal care services in this study. The mode of delivery, place of delivery, and receiving information about postnatal care during antenatal visits were significantly associated with postnatal care service utilisation.

**Conclusion:**

Less than half of the study participants were informed about postnatal care services, contributing to their low utilisation. Thus, it is vital to strengthen the provision of information, education, and communication with regard to postnatal care services among pregnant mothers visiting antenatal clinics.

## Introduction

The World Health Organisation (WHO) defines the postpartum period as the first six weeks (42 days) after delivery [1]. Postnatal care (PNC) refers to the care provided to the mother and the baby during this period. It encompasses a range of healthcare services designed to promote the health of women and newborn babies, ranging from risk identification, preventive measures, health education and promotion, as well as management and referral for complications. The postnatal period is a critical phase of life for both mothers and newborn babies as most maternal and child mortalities occur during this period [2]. Globally, maternal mortality remains high in certain regions and countries [3]. The Sustainable Development Goals (SDG) aims to reduce the maternal mortality rate to less than 70 per 100,000 live births by 2030 [3]. However, based on a recent report by the United Nations, maternal mortality due to complications of pregnancy or childbirth is still high even with a decline of approximately 38% from 342 deaths to 211 deaths per 100,000 live births between 2000 and 2017 [4]. However, these complications are still responsible for the death of approximately 810 women every day around the world. As high as 99% of these deaths occur in low and middle-income countries (LMICs) where conditions such as poverty, poor healthcare access, and suboptimal health systems are rampant with more than half of childbirths still taking place at home [5]. In addition, there are large disparities in maternal mortality between developing and developed countries, with 94% of the deaths occurring in LMICs due to lack of timely access to healthcare services [3].

Maternal healthcare, including postnatal care, directly affects the health of a mother and her newborn baby, hence greatly impacting maternal and neonatal morbidity and mortality [2]. The World Health Organisation (WHO) defines focused postnatal care as a four-schedule personalised care given to a woman immediately after delivery, within 48 h, two weeks, and six weeks [6]. A recent systematic review identified the essential components of effective PNC, including breastfeeding, immediate examination of mothers and babies, skin-to-skin care at birth for neonates, counselling for potential dangers of mothers and babies, immunisations for babies, and other services [7]. The WHO recommends that all women and newborn receive postnatal care in the first 24 h following childbirth, regardless of where the birth occurs, and subsequent postnatal check-ups in the first six weeks [1]. PNC seeks to improve maternal, newborn, and infant receiving essential postpartum, newborn care, and family planning services [8]. Therefore, PNC has been highly recommended in the WHO 2016–2030 global strategy for the improvement of women’s, children’s, and adolescents’ health [9]. Maternal deaths, as well as the risk of mortality for mothers and newborn babies, can be reduced by the provision of comprehensive PNC and antenatal care (ANC) [10]. Besides reducing maternal mortality, PNC is also vital in the prevention of long-term complications such as impairment and disabilities. PNC enables health professionals to identify postpartum problems so that prompt treatment can be instituted to prevent potential complications, thus safeguarding the health of the mother and baby. However, despite its critical importance, PNC remains the most neglected component in the continuum of maternal and childcare health provision. This was evidenced in the most recent Countdown report that highlighted a low coverage of PNC (45%) for mothers in LMICs [11].

The Yemen civil war, which began in early 2015 and is still raging on today, has led to one of the world’s worst humanitarian crises. This crisis affects all aspects of life, including healthcare. A total of 24 million people require assistance and millions are left without access to life-saving medical treatment and supplies, causing them to succumb to preventable diseases such as cholera, diabetes, and diphtheria. It also records one of the highest maternal mortality rates in the world, with 17% of deaths among reproductive-age females caused by childbirth complications. Even before the civil war began in 2015, pregnant women in Yemen struggled to access the healthcare services they need. Since the beginning of the civil war in Yemen, the maternal mortality rate has increased drastically from five deaths a day in 2013 to 12 deaths a day in 2019. The war has resulted in limited access to basic resources such as food and water. This is just one of the many main factors that compromise the health of millions of women and their newborn [12].

In addition, Yemen is one of the most impoverished countries in the world, ranked 177th on the Human Development Index. Poverty is a major contributing factor to poor maternal health in Yemen. Impoverished women lack the basic nutrition requirements, financial capacity, healthcare access, and sufficient education to ensure a safe delivery after they become pregnant. Women during childbirth and infants are particularly vulnerable during this health crisis, as adequate medical care throughout pregnancy and birth is essential. In Yemen, maternal healthcare services are almost free in all public healthcare facilities and are provided by skilled healthcare staff, including doctors, midwives, nurses, auxiliary midwives, and community health workers. However, despite the availability of free services, the civil war has dramatically decreased healthcare access across the nation. Almost half of the health facilities in the country are not functional as a result of the conflict. The remaining ones are understaffed, underfunded, and face medical equipment shortages, thus compromising the quality of care to ensure the wellbeing of women during pregnancy, childbirth, and after delivery [12].

According to a recent study [13], at least half of Yemeni women still gave birth at home and only one in five received a postnatal check-up. There is a clear lack of health awareness among new mothers, as evidenced in a study whereby the participants claimed that giving birth at home was a better option than a hospital and they perceived that postnatal check-up was only necessary in the event of complications requiring medical attention. With the ongoing civil war and failing healthcare system, the standard of maternal healthcare in Yemen is of the utmost concern. While maternal health, especially PNC, has never been universally accessible to all, its utilisation has now reached a critical point as a result of the Yemeni civil war [12]. Therefore, this study aimed to determine the utilisation of PNC in Yemen and the factors influencing the utilisation of PNC at primary healthcare facilities.

## Method

A descriptive cross-sectional study was conducted in five government healthcare centres in the Maeen District, Sana’a City, Yemen, i.e. Gaza, Maeen, Al Khair and Al Salam, Dila Hamedan, and Hani Twmar). All centres provide primary healthcare services and serve as referral centres. The study population targeted all mothers who attended child clinics with children between one and six weeks of age at the five centres. Out of the total study population of 1,932 patients, 321 postnatal mothers were conveniently sampled based on the criteria that they had children between 1 and 6 weeks and none of the children required urgent medical attention.

### Data collection

Data were collected from December 2022 to January 2023. After providing written informed consent, the participants completed a face-to-face semi-structured questionnaire in the local language (Arabic). All the collected data were checked by the researchers for completion.

The questionnaire captured sociodemographic characteristics of study participants (age, education level, family size, occupation, husband’s occupation, income, residence address), provider-related factors (number of pregnancies, mode of delivery, place of delivery, knowledge of attendance of PNC within the first six weeks after delivery, two-week PNC service utilisation, PNC services received by respondents, residence distance from the facility, reception at the health facility, teaching about postnatal care during ANC visits, and level of information received about PNC.

The English and Arabic versions of the questionnaire were validated by five experts to ensure that all questions were clearly worded and would not be misinterpreted. The Arabic version was used in the data collection. A pilot study was conducted before data collection and the reliability of the questionnaire was deemed acceptable (Cronbach’s alpha = 0.73).

### Data analysis

Data analysis was performed using the Statistical Package for the Social Sciences (SPSS 24.0). Descriptive statistics were used to describe frequency, percentage, mean, and standard deviation. Pearson’s chi-square test was used to find factors associated with PNC utilisation. Furthermore, binary multiple logistic regression analysis was used to identify significant predictors of PNC utilisation. Factors that were significant at *p* < 0.25 in the univariate analysis were entered in the multivariate analysis. Multivariate logistic regression with the backward elimination method was used to determine the predictors of PNC utilisation. A *p*-value ≤ 0.05 was considered statistically significant.

## Results

**Fig. 1:**
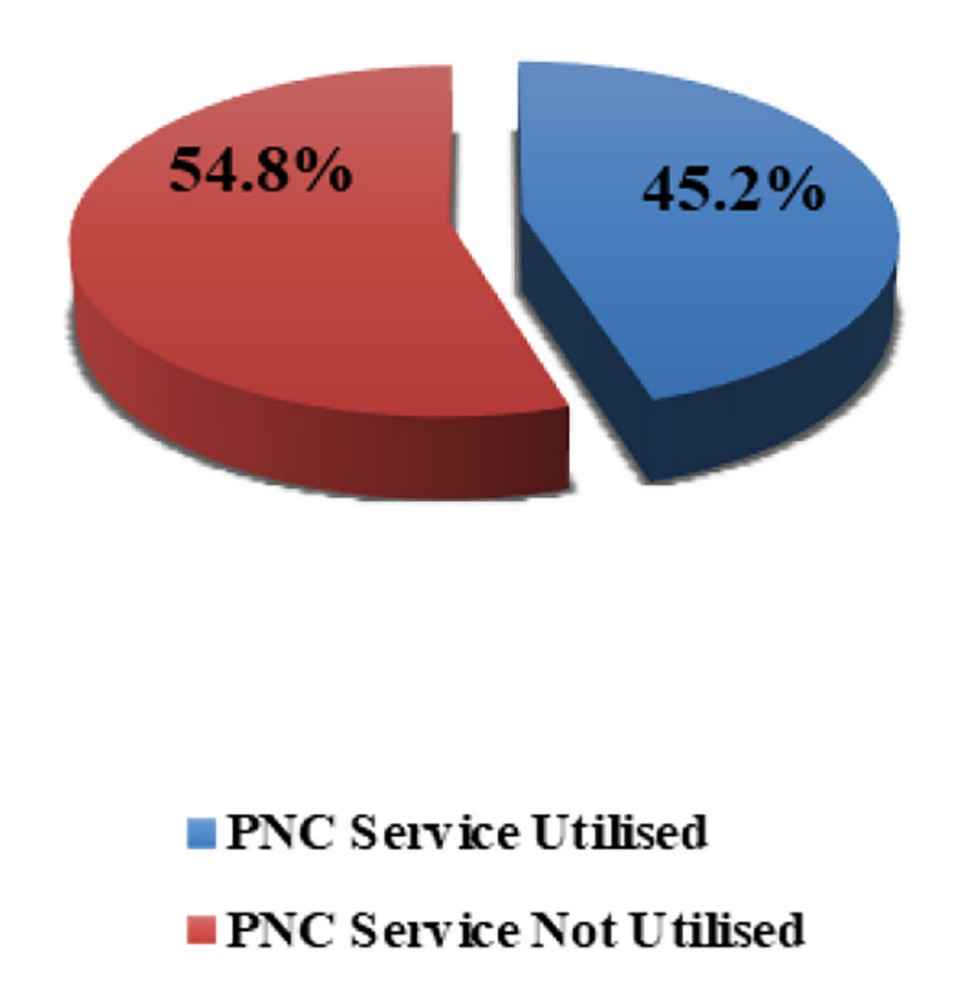
PNC service utilisation among study participants

More than one-third of the study participants were mothers between 21 and 25 years old. About one-third had completed primary school, while 34.9% of their husbands had completed secondary school. Less than half (40.5%) of them had 2–3 children. The majority (86.0%) of them were unemployed. Only 62.0% of their husbands were employed and 51.7% of them had insufficient family income. The majority (80.4%) of them lived in urban areas. In addition, three-quarters (75.4%) of them gave birth via normal vaginal delivery (Table 1). Figure 1 demonstrates that more than half 176 (54.8%) of them did not utilise PNC.

**Table 1:** Sociodemographic characteristics of maternal

Variable	Category	F	%
**Age**	16-20yrs	50	15.6
	21-25yrs	110	34.3
	26-30yrs	81	25.2
	30-35yrs	50	15.6
	More than 35yrs	30	9.3
**Maternal education**	Illiterate	69	21.5
	Primary school	103	32.1
	Secondary school	92	28.7
	Above secondary	57	17.8
**Husband education**	Illiterate	22	6.9
	Primary school	86	26.8
	Secondary school	104	32.4
	Above secondary	109	34.0
**Number of children**	1	97	30.2
	2–3	130	40.5
	4–5	59	18.4
	More than 5	35	10.9
**Maternal occupation**	Employment	45	14.0
	Non employment	276	86.0
**Husband occupation**	Employment	199	62.0
	Nonemployment	122	38.0
**Family income**	Enough	155	48.3
	Not enough	166	51.7
**Residence place**	Urban	258	80.4
	Rural	63	19.6
**Number of pregnancies group**	1	77	24.0
	2–3	140	43.6
	4–5	51	15.9
	More than 5	53	16.5
**Mode of delivery**	Normal delivery	265	82.6
	Cesarean section	56	17.4

Table 2 revealed that almost three-quarters (73.8%) of the study participants delivered in the healthcare facilities during their most recent pregnancies and 60.1% of them were discharged after 24 h. Only one in five (19.6%) attended ANC and had some knowledge of PNC services within the first six weeks after delivery. The majority (65.4%) of them attended PNC services within two weeks. However, 52% of maternal were unaware of PNC services. Most of them (64.8%) came for childhood immunisation. About half (53.3%) received information about PNC services from healthcare professionals. In addition, most of them (93.x%) resided 0–5 km from the nearest healthcare facility and 57.6% perceived a friendly reception upon visit. However, half of them (52.6%) did not receive any information on PNC during ANC visits.

**Table 2:** Maternal sociodemographic characteristics

Variable	Category	F	%
**Age**	16-20yrs	50	15.6
	21-25yrs	110	34.3
	26-30yrs	81	25.2
	30-35yrs	50	15.6
	More than 35yrs	30	9.3
**Maternal education**	Illiterate	69	21.5
	Primary school	103	32.1
	Secondary school	92	28.7
	Above secondary	57	17.8
**Husband education**	Illiterate	22	6.9
	Primary school	86	26.8
	Secondary school	104	32.4
	Above secondary	109	34.0
**Number of children**	1	97	30.2
	2–3	130	40.5
	4–5	59	18.4
	More than 5	35	10.9
**Maternal occupation**	Employment	45	14.0
	Non employment	276	86.0
**Husband occupation**	Employment	199	62.0
	Non employment	122	38.0
**Family income**	Enough	155	48.3
	Not enough	166	51.7
**Residence place**	Urban	258	80.4
	Rural	63	19.6
**Number of pregnancies group**	1	77	24.0
	2–3	140	43.6
	4–5	51	15.9
	More than 5	53	16.5
**Mode of delivery**	Normal delivery	265	82.6
	Cesarean section	56	17.4

Table 3 displays the bivariate analysis between the maternal and healthcare-related characteristics with PNC utilisation. All the factors with a *p*-value < 0.25 (age, maternal and husband education, number of children, maternal and husband occupation, family income, residence place, place and mode of delivery, attendance of the first medical assessment, knowledge of attendance of postnatal care service within the first six weeks after delivery, postnatal services received by respondents, awareness of postnatal care services, information on PNC services, residence distance from the health facility (kilometres, km), reception at the health facility, teaching about PNC during ANC visits, and level of information received on PNC) were inserted into the multivariate logistic regression model. Following that, significant predictors of PNC service utilisation were identified. Mothers who lived in urban places were approximately four times (OR = 4.033, 95% CI: (1.268–12.826) more likely to utilise PNC than those who lived in rural places; mothers who delivered their last child at home were approximately five times (OR = 4.521, 95% CI: [0.984–20.776]) more likely to utilise PNC than those who delivered in health facilities; mothers who were taught about postnatal care during ANC visits were approximately 12 times (OR = 12.395, 95% CI: [4.663–32.948]) more likely to utilise PNC services (Table 4).

**Table 3:** Risk factors associated with PNC service utilisation among maternal using binary logistic regression

Independent Variables	B	S.E	Wald	*p*-value	AOR	95%	CI
**Constant**	-5.998	3.191	3.534	0.060	0.002		
**Residence place**	1.743	0.640	7.419	0.006	5.712	1.630	20.018
**Place of delivery**	1.258	0.720	3.051	0.081	3.517	0.858	14.422
**Teaching about postnatal care during ANC visits**	1.472	1.150	1.639	0.000	4.357	0.458	41.474

**Table 4:** Risk factors associated with PNC service utilisation among maternal using multivariate logistic regression

Variable	Category	Utilised PNC		COR(95% CI)
		Yes	No	
**Residence place**	Urban	123	135	4.033(1.268–12.826)
	Rural	22	41	1
**Place of delivery**	Home	23	61	1
	Health facility	122	115	4.521(0.984–20.776)
	No	22	79	1
**Teaching about postnatal care during ANC visits**	Yes	99	53	12.395(4.663–32.948)
	No	46	123	1

## Discussion

This study aimed to identify the factors affecting the utilisation of PNC among mothers who attended primary health centres in Yemen. In this study, of the total 321 participants, one-third of them (34.3%) of the participants were considerably young, aged between 21 and 25 years old. This was in contrast with a study in Kenya by Mayieka et al. [14] that included a higher proportion (69.7%) of the younger age group (21–30 years old) in their study. Apart from that, Salunkhe & Katti [15] found that half of their participants (51.7%) were aged 23–27 years old. Dhakal et al. [16] mentioned that most women who had their first pregnancy in Nepal were 20 to 24 years old. As for marital status, almost all of the participants in this study (96.9%) were married. This was aligned with Lwelamira et al. [17], Njoka et al. [18], and Chungu et al. [19] whereby 73.9%, 75.7%, and 80% of the study participants were married. In the present study, 40.5% of participants had 2–3 children while 30.2% had only one child. This was in contrast with Njoka et al. [18] that reported more than half (53.9%) of their study participants had 2–3 children. Similarly, Dairo & Atanlogun [20] in the southwestern region of Nigeria reported that less than half (47.9%) of the participants had given birth to only one child while Mayieka et al. [14] mentioned that 45.5% of their participants had 2 or 3 children.

Apart from that, one-third of the study participants in this study (32.1%) had primary education as compared to 34.9% of their husbands with secondary school education levels. In contrast, Dhakal et al. [16] included 50% of illiterate women while Chungu et al. [19] had half of the study participants (54%) with a primary school education level. In Mashhad, Iran, Mirzaii et al. [21] reported that most of their study participants had a high school diploma (53.2%).

In addition, the majority (86.0%) of the participants in this study were unemployed, similar to the studies in Iran by Mirzaii et al. [21] and in Gaza by Al-Masri [22] that included mostly housewives (94% and 96.8%). As for their spouses, 62.0% of the husbands were employed in this study, in line with Al-Masri et al. [22] with 71.2% of husbands being employed. On the other hand, Mayieka et al. [14] found that 65.4% of participants were unemployed. In line with the employment status, about half (51.7%) of the participants in this study did not have enough income. This percentage of financial constraint was higher than Limenih et al. [23] whereby only 38.4% of the mothers in the north western region of Ethiopia had an average monthly income of less than 25 dollars. Similar findings are reported in Bhai District, Tanzania [17] with as high as 84.3% of the participants coming from households of low income. As for the area of residence, four out of five (80.4%) of them were living in urban areas, similar to a study in the Kampala District by Christine & Kizito [24] whereby half (52.5%) of the participants resided in town areas. Another study in Akufo City by Naji et al. [25] also showed that more than half of the participants (53.5%) were living in urban areas.

In this study, 45.2% of the participants utilised PNC services. Similar levels of utilisation were reported in the Kamba area (45.1%) [18] and Aysaeta District (45.1%) [26]. The low uptake might be attributed to many reasons, including poor compliance, long distance from healthcare facilities, low family income, unpleasant experience with midwives in previous delivery, lack of awareness about PNC, inadequate services in the health facility, cost of service and transportation, and husbands’ refusal to bring their wives to health facilities for delivery due to low educational level. The level of PNC utilisation in this report was lower compared to previous studies conducted in Bangladesh (63%) [27], Thailand (70.7%) [28], Uganda (58%) [29], Bahi District, Tanzania (71.6%) [17], Kenya (57%) [14], and Zambia (63.4%) [19]. In contrast, the PNC utilisation rate in this study is considerably higher than in some other African countries, such as in the Ethiopian East Gojjam Zone (34.6%) [30], Wolkite town of Gurage Zone (23.3%) [31], Goba Woreda of Bale Zone (42.2%) [32], District Arsi Zone (23.7%) [33], Debre Markos Town (33.5%) [23], Dessie Town (37%) [34], Nigeria (5.8%) [20], and Kampala District, Uganda (14%) [24]. Additionally, it was also higher than Nepal (34%) [16] and Palestine (36.6%) [35]. On the other hand, the rate of PNC utilisation varied in several Ethiopian studies, including those in Halaba Kulito Town (47.9%) [36], Belabor Town (57.5%) [37], Yirgalem Town of Sidama Regional State (66.7%) [38], Shebe Sombo Woreda of Jimma Zone (58.5%) [39]. Some of the variations in PNC utilisation could be attributed to underlying causes such as differences in culture, socioeconomic levels, demographic diversity, and governmental political concerns. Women in certain LMICs might not have a high degree of knowledge of the availability and importance of PNC. Furthermore, pre-existing cultural and spiritual taboos as well as the traditional beliefs and ways of life of different communities also have a role to play. These societal and cultural factors may have prevented women from seeking medical attention, especially if they are given the authority to be the decision-makers.

In this study, more than two-thirds (73.8%) of the participants delivered their last baby in a health facility. These results were in agreement with Ishak et al. [40] and Wordofa et al. [32] who reported a high percentage of mothers (92% and 73% respectively) who delivered in a health facility with the help of skilled providers. Our study shows that less than half (43.6%) of the participants had 2–3 pregnancies, as compared to a study conducted in Northern Shewa, Ethiopia by Angore et al. [41] in which the majority of the women (55.1%) had 2–3 pregnancies. Moreover, three-quarters (75.4%) of our participants gave birth via spontaneous vaginal delivery, closely similar to Angore et al. [41] which reported 87.4% of spontaneous vaginal deliveries. Sa et al. [34] mentioned that 72.9% of participants in northern Ethiopia delivered by spontaneous vaginal delivery while Wudineh et al. [37] reported that 77.3% of participants in Depredator Town, Northwest Ethiopia delivered by spontaneous vaginal delivery.

With regard to PNC, 30.2% of the participants in the study visited the health facilities for childhood immunisation services, in line with Wudineh et al. [37] and Peter & Kinuthia et al. [42] that reported 50% and 38.2% of participants who attended the clinics for child immunisation services. However, almost half (52%) of participants in this study had no awareness about PNC services. The awareness level is also low in a study by Thaulo & Kambala [43] whereby 35.6% of the women in rural Malawi had low knowledge concerning PNC services. Njoka et al. [18] also found that most women did not attend PNC services because they lacked awareness of PNC services (69.1%).

In terms of distance, the majority (93%) of participants in this study lived 5 km from the nearest health facility, similar to Peter & Kinuthia [42] whereby all women resided less than 5 km from the nearest hospital. In another study by Gborgbortsi et al. [44] in the Eastern Region, Ghana, the median expected travel distances to secondary and primary facilities were even nearer at 3 km. Additionally, most of the study participants perceived that they were given a friendly reception at the health facility they attended. These findings align with Mayieka et al. [14] in which most participants (65.7%) stated a good reception at the health facility too. Another significant predictor of PNC utilisation was having a residence in urban areas, rather than rural areas. This finding is similar to a study conducted in the Aysaeta District, Northeast Ethiopia by Ibrahim et al. [26], as well as in Nepal [45], Zambia [46], and Kakamega [47]. Women who live in urban regions often have better access to PNC services as they have more exposure to health promotion initiatives. In contrast, those who reside in rural areas face more barriers to attending health facilities, given the distance, lack of transportation, as well as traditional cultures and customs that make it difficult to use delivery services. A low uptake of PNC is more likely to predispose to morbidity and mortality [26].

Apart from that, our study also revealed that PNC service utilisation among mothers who gave birth at health facilities was five times higher than those who had home deliveries. This finding is consistent with studies in Ethiopia [34, 37], Tanzania [17, 48], Nigeria [49], and Bangladesh [27], all of which reported a significant association between delivery in health facilities and subsequent PNC utilisation. One of the likely explanations was the access to information and learning about PNC services and their advantages among women giving birth in a medical facility. On a similar note, mothers who were taught about PNC during ANC visits were approximately 12 times more likely to utilise it. This finding is in agreement with a study conducted in Kenya by Mayieka et al. [14]. Close attention given by health professionals, health managers, and policy makers to PNC follow-up programmes, as well as adequate monitoring and evaluation within the healthcare institution can boost the uptake of PNC. For instance, counselling about PNC and referrals by health professionals can empower mothers to take up the decision-making authority at the household level to enhance PNC utilisation.

Last but not least, nursing or midwifery staff play an important role in educating mothers about PNC services during the first to the sixth week after delivery as part of providing responsive care for their newborn. They must counsel and teach new mothers about the importance of the first few weeks to establish healthy relationships and behaviours that can enhance long-term infant development and health. In addition, they must encourage mothers to utilise PNC services such as breastfeeding counselling to ensure the correct positioning of breastfeeding and optimal mother-baby attachment. By providing a positive healthcare experience where people are treated with dignity and respect, they can support the active participation of mothers and patients in healthcare decisions.

## Conclusion

PNC utilisation remains low in Yemen. This study highlights that residence, place of delivery, and teaching about PNC during ANC visits are vital determinants of PNC service utilisation. Maternal healthcare service providers play an important role in enhancing the uptake of PNC services by providing information, education, counselling, and communication for mothers at the community and facility levels. In view of this, the local government agencies must collaborate to improve awareness and institute behavioural changes, especially in rural areas.

## Data Availability

The datasets used and/or analysed during the current study are available from the corresponding author upon reasonable request.

## Abbreviations

WHO: World Health Organisation
ANC: Antenatal care
PNC: Postnatal care
SPSS: Statistical package for the social sciences
SD: Standard deviation
COR: Crude odds ratio
AOR: Adjusted odds ratio
CI: Confidence interval
